# The Ricinus Communis as an Emmenagogue and Galactagogue

**Published:** 1855-04

**Authors:** 


*The Ricinus Communis as an Emmenagogue and Galactagogue.*—Clara Shymaski, brought to the Charity Hospital 25th December, 1853, bed 522, ward 34; the following was gathered by Dr. Nott, physician to the ward: She had had amenorrhœa for six months, with vomiting of blood at short intervals; wandering pains throughout the whole body, and a fixed pain in the right hypochondriac region. The skin around the margin of the umbilicus was of a reddish hue, and from the centre there exuded a milky fluid, which she has observed for nearly three years; she had dull pains in the temple recurring frequently; insomnia with nightmare, which caused much disquietude; but little appetite. The patient had yellow fever in 1853, at the same time as her husband, whom she was unfortunate enough to lose; to grief at this bereavement, together with the privations which were consequent, and to bad medical treatment for subsequent mental and physical disorders, may be traced the chain of symptoms, of which the above is a synopsis.

After three days' treatment directed to the relief of the most urgent symptoms, Dr. Nott orderered the following: four or five ounces of the leaves of the *palma christi* (*ricinis communis*) to be boiled in six or seven pints of water; with this decoction: the breasts to be rubbed, for ten or twenty minutes three times daily, afterwards the leaves applied to the parts and permitted to dry, the treatment to be continued for three days, when if no good result followed, to be abandoned as useless.

On the second day of treatment a few drops of menstrual blood appeared; on the third, the menstrual flow occurred and lasted for several days; the patient after this experienced great relief, and was better than she had been for a long time; her appetite returned, together with good spirits. She left the hospital on the 19th of January, completely well.

It may be well to remark, that during the application of the remedy, the patient experienced sharp pains in the breasts, which, however, disappeared after its discontinuance.

Margurite Blans, aged 18, arrived in this city from Havre, towards the end of October, 1854; was brought to the Charity Hospital on the 15th December; sick eight days with typhoid fever with pulmonary symptoms. The disease progressed and assumed a grave character, great prostration, with very feeble pulse, no appetite, much thirst, indisposition to converse, oppressive pain in the chest notwithstanding a diminution of the pulmonary engorgement; there was great doubt as to the termination of the disease, when I discovered by questioning her, that she had not menstruated since her arrival in New Orleans; this fact was communicated to Dr. Nott, who ordered a trial of the treatment mentioned in the first case. The results were equally happy; on the third day the menses began and lasted for several days. Her health rapidly improved, her appetite re-appeared, and in a short time she regained sufficient strength to leave the hospital, after a sojourn of one month.

There are two varieties of the plant, the white and red; the latter is carefully avoided, as it is a violent irritant and produces an inordinate flow of the menses.—*New Orleans Medical News and Hospital Gazette.*

